# Diabetes mellitus in older persons with neurocognitive disorder: overtreatment prevalence and associated structural brain MRI findings

**DOI:** 10.1186/s12877-024-05025-x

**Published:** 2024-05-14

**Authors:** Pauline Putallaz, Laurence Seematter-Bagnoud, Bogdan Draganski, Olivier Rouaud, Hélène Krief, Christophe J. Büla

**Affiliations:** 1 Service of geriatric medicine and geriatric rehabilitation, University of Lausanne Medical Center (CHUV), Route de Mont Paisible 16, Lausanne, 1011 Switzerland; 2 Service of geriatric medicine, Hospital of Valais, Avenue de la Fusion 27, Martigny, 1920 Switzerland; 3 Department of Epidemiology and Public Health (Unisanté), Lausanne, 1011 Switzerland; 4 Laboratory of Research in Neuroimaging (LREN) - Department of Clinical Neuroscience - CHUV, University of Lausanne, Lausanne, 1011 Switzerland; 5 Leenaards Memory Center, University of Lausanne Medical Center (CHUV), Route de Mont Paisible 16, Lausanne, 1011 Switzerland

**Keywords:** Diabetes mellitus, Cognitive impairment, Dementia, Alzheimer’s disease, Neuroimaging, Voxel-based morphometry, Hypoglycemia, Older patients

## Abstract

**Background:**

Tight diabetes control is often applied in older persons with neurocognitive disorder resulting in increased hypoglycemic episodes but little is known about the pattern of brain injury in these overtreated patients. This study aims to: (a) quantify the prevalence of diabetes overtreatment in cognitively impaired older adults in a clinical population followed in an academic memory clinic (b) identify risk factors contributing to overtreatment; and (c) explore the association between diabetes overtreatment and specific brain region volume changes.

**Methods:**

Retrospective study of older patients with type 2 diabetes and cognitive impairment who were diagnosed in a memory clinic from 2013 to 2020. Patients were classified into vulnerable and dependent according to their health profile. Overtreatment was defined when glycated hemoglobin was under 7% for vulnerable and 7.6% for dependent patients. Characteristics associated to overtreatment were examined in multivariable analysis. Grey matter volume in defined brain regions was measured from MRI using voxel-based morphometry and compared in patients over- vs. adequately treated.

**Results:**

Among 161 patients included (median age 76.8 years, range 60.8–93.3 years, 32.9% women), 29.8% were considered as adequately treated, 54.0% as overtreated, and 16.2% as undertreated. In multivariable analyses, no association was observed between diabetes overtreatment and age or the severity of cognitive impairment. Among patients with neuroimaging data (*N* = 71), associations between overtreatment and grey matter loss were observed in several brain regions. Specifically, significant reductions in grey matter were found in the caudate (adj β coeff: -0.217, 95%CI: [-0.416 to -0.018], *p* = .033), the precentral gyri (adj βcoeff:-0.277, 95%CI: [-0.482 to -0.073], *p* = .009), the superior frontal gyri (adj βcoeff: -0.244, 95%CI: [-0.458 to -0.030], *p* = .026), the calcarine cortex (adj βcoeff:-0.193, 95%CI: [-0.386 to -0.001], *p* = .049), the superior occipital gyri (adj βcoeff: -0.291, 95%CI: [-0.521 to -0.061], *p* = .014) and the inferior occipital gyri (adj βcoeff: -0.236, 95%CI: [-0.456 to – 0.015], *p* = .036).

**Conclusion:**

A significant proportion of older patients with diabetes and neurocognitive disorder were subjected to excessively intensive treatment. The association identified with volume loss in several specific brain regions highlights the need to further investigate the potential cerebral damages associated with overtreatment and related hypoglycemia in larger sample.

**Supplementary Information:**

The online version contains supplementary material available at 10.1186/s12877-024-05025-x.

## Background

Type 2 diabetes and dementia are epidemiologically linked, with both conditions showing increased incidence and prevalence as populations ages. For instance, results from a study in general practice showed that out of one hundred patients with diabetes, only two suffered from dementia, but out of one hundred patients with dementia, as much as one in six also suffered from diabetes [1]. The pathophysiological mechanisms underlying this association are still debated [2, 3]. In individuals with type 2 diabetes, cognitive function may be influenced not only by direct disease mechanisms but also by an array of comorbidities and lifestyle factors. These include hypertension, dyslipidemia, cardiovascular diseases, obesity, and physical inactivity, as well as psychological factors like depression and the risk of iatrogenic hypoglycemia. Moreover, the management of diabetes plays a crucial role, where chronically high levels of glycated hemoglobin (HbA1c) and a longer duration of the condition have been linked to accelerated cognitive decline [4–6]. Macro- and microvascular lesions, endothelial dysfunction resulting in increased permeability of the blood-brain barrier, direct glucotoxicity as well as alterations in intracerebral insulin signaling pathways all lead to hypoxemia, inflammation, and neuronal death [7]. Indeed, some of these mechanisms have also been reported in Alzheimer’s disease [8, 9]. Several studies are currently underway to investigate the potential neuroprotective effect of antidiabetic treatments with a growing enthusiasm for new molecules such as GLP-1 analogues [10] and SGLT-2 inhibitors [11] or older ones like metformin [12].

However, several studies suggest that hypoglycemia could in turn increase the risk of neurocognitive disorders [13]. Indeed, large reductions in HbA1c have been associated with an increased risk of dementia in older adults with type 2 diabetes [14]. Moreover, intensive glycemic control doubles the risk of severe hypoglycemia according to results of a meta-analysis of four major trials (ACCORD, ADVANCE, UKPDS, and VADT) [15]. Older patients and those with multimorbidity are at most increased risk of hypoglycemia according to a large US cohort study [16]. In particular, patients with diabetes and dementia appear at especially increased risk of hypoglycemia [13, 17].

Over the last 10 years, guidelines advocated for the customization of diabetes treatment according to patient’s health status, i.e., whether robust (in good health, few comorbidities), vulnerable (mild functional or cognitive impairment, or multiple comorbidities), or dependent (poor health, limited life expectancy) [18–20]. The ramifications of these adapted recommendations are complicated to know to what extent they are unequivocally determined. While many studies still report that a significant proportion of older patients with diabetes remain potentially overtreated [21–23], others suggest a trend toward deintensification of diabetes treatment. For instance, a recent study conducted among patients from memory clinics in Japan reported a decrease from 2012 to 2020 in the proportion of overtreated patients and in the utilization of treatment with a high risk of hypoglycemia [24]. Whether these observations are similar in other countries remains however uncertain.

An additional question relates to whether hypoglycemia induces specific structural brain damages in patients with type 2 diabetes. For instance, in a cohort with a prolonged follow-up (1987-89 to 2011-13), hypoglycemia was associated with smaller volume of the total brain and the prefrontal region [25]. Inversely, another study that used repeated magnetic resonance imaging (MRI) over 40 months reported slightly less brain atrophy among individuals who had at least one severe hypoglycemic episode (glycemia < 2.8mmol/l or requiring assistance) [26].

The present study was undertaken to get further insight into the relationship between type 2 diabetes overtreatment in older people and potential brain damages among patients with neurocognitive disorder. In a cohort of older patients with type 2 diabetes and neurocognitive disorder who consulted a memory clinic, the main objective was (a) to investigate the proportion of patients potentially overtreated according to their health profile, (b) determine factors associated with diabetes over- or undertreatment, respectively and (c) investigate differences in brain area volume associated with diabetes overtreatment. Our hypothesis was that overtreatment would be associated with grey matter volume loss in specific areas, e.g., hippocampus and basal ganglia, due to a greater sensitivity to hypoglycemia in these regions [27].

## Methods

### Setting and population

This retrospective observational study enrolled patients who consulted the Leenaards Memory Center [28] at the Lausanne University Hospital from January 2013 to November 2020. Patients included in the main analysis were (a) those aged 60 years or more; (b) diagnosed with a neurocognitive disorder with a Clinical Dementia Rating (CDR) ≥ 0.5; (c) with a diagnosis of type 2 diabetes and treated with at least one antidiabetic medication; (d) with HbA1c documented in their electronic health record (EHR) within 3 months before or 12 months after the consultation; (e) who did consent to the use of routinely collected data for retrospective research. Robust patients, corresponding to “Healthy” according to *the American Diabetes Association’s* (ADA), were excluded, as one criterion is a CDR < 0.5.

For the neuroimaging analysis, patients were further selected if they had a documented MRI within 3 weeks before or 18 months after the consultation. Patients considered as undertreated were excluded from this subgroup analysis.

The study was approved by the Cantonal Commission on Ethics in Human Research (IP 2020 − 01615).

### Data source

Since 2013, clinical data pertaining to patients’ visits at the Leenaards Memory Center are prospectively collected in a specific registry (Cohort Leenaards Memory and Neurosciences (CLEMENS)). Data on age, gender, cognitive diagnoses, neuropsychological status, CDR, and MRI morpho-volumetric data were retrieved from this registry by the data manager for patients who visited the center within the defined time frame.

Additional data were retrieved from patients’ EHR by a single investigator: living arrangement (living alone or not), type of diabetes, antidiabetics treatment, HbA1c value (within the defined time-window), number and type of comorbidities (among the following: arthritis, cancer, heart failure, depression, emphysema, falls, hypertension, incontinence, stage 3 or worse chronic kidney disease, history of stroke or heart attack), functional performance in basic [29] and instrumental [30] activities of daily living (BADL and IADL, respectively). Comorbidities were considered only if serious enough to warrant medication or lifestyle adjustments (dietary changes, physical activity, and modest weight loss (e.g., 5–7%)), as proposed by the *ADA* [18].

### Definitions

#### Health profile

Patients’ health profiles were classified into two categories based on the *ADA’s 2020* guidelines: ‘Vulnerable’ (mild functional or cognitive impairment, or multiple comorbidities) and ‘Dependent’ (significant functional or severe cognitive impairment). Classification was performed by the principal investigator (P.P) and validated by a senior co-investigator (L.S-B).

- ***Vulnerable (corresponding to “Complex/Intermediate” according to the ADA)***: mild functional decline (Lawton’s IADL < 6/8) or mild cognitive impairment (CDR ≥ 0.5) or at least 3 comorbidities.
- ***Dependent (corresponding to “Very Complex/Poor Health” according to the ADA)***: terminal illness or significant functional impairment (Katz’s BADL < 4/6) or moderate to severe cognitive impairment (CDR ≥ 2).

#### Diabetes treatment

Diabetes treatment was evaluated as over-, under-, or adequately treated, aligning with the patient’s health profile and HbA1c targets, following the latest guidelines [18–20]. Specific HbA1c targets were set for vulnerable (HbA1c target between 7 and 8%) and dependent patients (HbA1c target between 7.6% and 8.5%), with treatments classified by hypoglycemia risk (*high risk of hypoglycemia* medication were insulin therapy, sulfonylureas and glinides [20]).

A sensitivity analysis was performed using the “Choosing Wisely” definition of overtreatment (i.e., “Reasonable glycemic targets would be 7.0–7.5% in healthy older adults with long life expectancy, 7.5–8.0% in those with moderate comorbidity and a life expectancy < 10 years, and 8.0–9.0% in those with multiple morbidities” [31]).

Diabetes treatment was further assessed as *potentially inappropriate* according to the following criteria [20, 31, 32]:

1. Using a medication other than metformin to achieve a target HbA1c < 7.5%, according to the “Choosing Wisely” definition.
2. metformin, gliclazide, glimepiride and glibenclamide if estimated glomerular filtration rate (GFR) < 30 ml/min.
3. insulin in case of cognitive impairment with CDR ≥ 1 unless formal and/or informal home support for administration / supervision is secured.
4. GLP-1 analogues if body mass index (BMI) ≤ 18.5 kg/m^2^ or undernutrition is documented in the EHR.
5. glifozine in patients with a history of falls, recurrent urinary tract infection, urinary incontinence, chronic alcohol abuse, or if the estimated GFR < 45 ml/min, or GFR < 30 ml/min in the presence of congestive heart failure.

### Neuroimaging data

Anonymized clinical T1-weighted MRI data, acquired at 1.5T and 3T scanners (Siemens, Erlangen Germany) for diagnostic purposes, were used. We estimated individuals’ grey matter volume from the available T1-weighted MRI data using SPM12s (Wellcome Centre for Human Neuroimaging, UCL, London, UK) multi-channel “unified segmentation” [33] with enhanced tissue priors that provide superior detection of thalamus and basal ganglia [34] running under MATLAB_R2021 (MathWorks, Sherbon, MA). The sum of grey matter, white matter and cerebro-spinal fluid represented the total intracranial volume. Finally, we calculated 127 regional averages of volume values across cortical and subcortical grey matter areas using the factorisation-based image labelling [35]. To mitigate partial volume effects during segmentation, a uniform threshold of 20% was applied. Averages were calculated for bilateral regions, detailed further in the appendix.

The evaluation of leukoencephalopathy was performed based on radiologists’ reports from MRI or CT-scan assessments, categorizing the condition as mild (Fazekas 1), moderate (Fazekas 2), or severe (Fazekas 3).

### Statistical analyses

The characteristics of under-, adequately, and overtreated patients were compared in bivariable analysis using Chi-square test for categorical variables or Fisher exact test in case of groups of 5 persons or less. We used mean comparison tests in case of continuous variables, Kruskal-Wallis or ANOVA (analysis of variance) for analyses with a result in > 2 categories.

To identify the characteristics independently associated to over- or undertreatment, a multinomial regression model was performed, adjusting for variables significantly associated in bivariable analysis.

Grey matter volume in defined brain regions was compared in patients overtreated or adequately treated, using the regional volume averages from the T1-weighted MRI. For each brain region, multivariable logistic regression models were employed, with adjustments made for age, sex, exposure to treatments associated with a high risk of hypoglycemia, and the presence of hypertension.

All analyses were repeated using the “Choosing Wisely” criteria, as sensitivity analysis.

For the statistical analyses we used the STATA software, version 16.

## Results

**Fig. 1:**
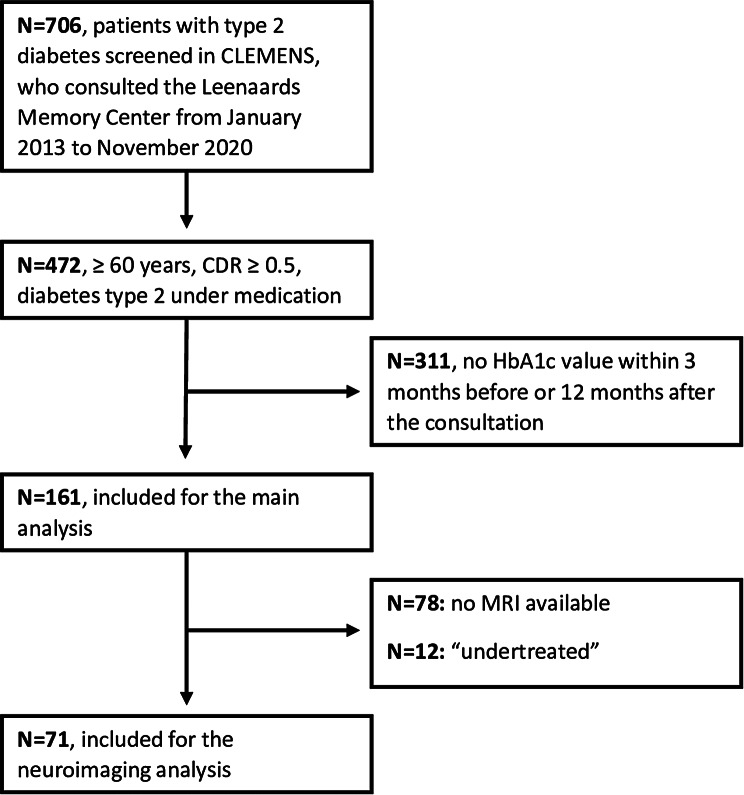
Flowchart

We screened *N* = 706 patients with type 2 diabetes in CLEMENS who consulted the Leenaards Memory Center from January 2013 to November 2020. Among them, *N* = 472 met the inclusion criteria of age > 60 years, under antidiabetic medication and with a CDR ≥ 0.5. HbA1c value (within 3 months before or twelve months after the consultation) was missing in *N* = 311 patients (they were more likely to be female and to have Alzheimer’s disease [see appendix]). Thus, a total of 161 patients met the inclusion criteria for the main analysis and were analyzed (Fig. 1).

Most patients were Caucasian and lived at home (data not shown). Median age was 76.8 years (range 60.8–93.3 years), about two-thirds were male (67.1%), more than half (55.9%) had at least 3 comorbidities, but only 6.8% had a significant functional dependency, defined as Katz’s BADL score < 4 **(**Table 1**)**. Most patients suffered from mild dementia (CDR ≤ 1), mainly from probable Alzheimer’s disease and vascular dementia.

**Table 1:** Bivariable analysis comparing the characteristics of under-, adequately and over-treated patients

		Treatment adequation			
	**All** N = 161 (100.0%)	**Undertreated** N = 26 (16.2%)	**Adequate** N = 48 (29.8%)	**Overtreated** N = 87 (54.0%)	p-value*
Female Sex	53 (32.9)	7 (26.9)	17 (35.4)	29 (33.3)	.754
Age					.779
Median [range]	76.8 [60.8–93.3]	75.6 [62.7–86.2]	76.5 [61.5–93.3]	77.0 [60.8–91.6]	
Health status					.165
Vulnerable	135 (83.9)	25 (96.2)	39 (81.3)	71 (81.6)	
Dependent	26 (16.2)	1 (3.9)	9 (18.8)	16 (18.4)	
Living alone	54 (33.8)	12 (46.2)	14 (29.2)	28 (32.6)	.317
Homecare present	61 (38.4)	14 (56.0)	14 (29.2)	33 (38.4)	.082
≥ 3 comorbidities	90 (55.9)	20 (76.9)	25 (52.1)	45 (51.7)	.062
Katz Basic ADL^†^ <4	11 (6.8)	1 (3.9)	3 (6.3)	7 (8.1)	.915
Lawton Instrumental ADL ^‡^ <6	70 (43.5)	12 (46.2)	20 (41.7)	38 (43.7)	.932
Etiology of the cognitive disorder					.973
Alzheimer’s disease	52 (32.3)	7 (26.9)	17 (35.4)	28 (32.2)	
Vascular dementia	65 (40.4)	11 (42.3)	18 (37.5)	36 (41.4)	
Other	43 (26.7)	7 (26.9)	13 (27.1)	23 (26.4)	
Missing	1 (0.6)	1 (3.8)	0 (0)	0 (0)	
CDR ^§^ >0.5	66 (41.0)	10 (38.5)	17 (35.4)	39 (44.8)	.545
Leukoencephalopathy					.015
Moderate/severe	67 (41.6)	17 (65.4)	21 (43.8)	29 (33.3)	
Light	85 (52.8)	8 (30.8)	24 (50.0)	53 (60.9)	
Missing	9 (5.6)	1 (3.8)	3 (6.3)	5 (5.7)	
Insulin in the treatment	46 (28.6)	21 (80.8)	14 (29.2)	11 (12.6)	< .001
Treatment with a high risk of hypoglycemia	69 (42.9)	24 (92.3)	25 (52.1)	20 (23.0)	< .001

* p-value from Pearson chi-squared test or Fisher exact test (categorical variables), and Student’s t-test or Wilcoxon’s test (continuous variables)

*† Katz Basic Activities of Daily Living (BADL)*: includes bathing, dressing, going to the WC, transferring, maintaining continence, eating, score ranges from 0 to 6 with higher scores indicating better function [29]

*‡ Lawton Instrumental Activities of Daily Living (IADL)*: includes using the phone, managing the finances, managing the medication, preparing meals, doing the laundry, cleaning, shopping, and using the transportation; Score range from 0 to 8 with higher scores indicating better function [30]

*§ CDR*: calculated on the basis of six different cognitive and behavioral domains such as memory, orientation, judgment and problem solving, community affairs, home and hobbies performance, and personal care. Scale of 0–3: no dementia (CDR = 0), very mild cognitive impairment (CDR = 0.5), mild dementia (CDR = 1), moderate (CDR = 2), and severe dementia (CDR = 3) [53]

Few (30/161, 18.6%) patients received one or more medications considered as potentially inappropriate and most of them because of the first criteria i.e. “Using a medication other than metformin to achieve a target HbA1c < 7.5%”, according to the “Choosing Wisely” definition (26 patients) [31]. In contrast, 42.9% of the patients had a medication with a high risk of hypoglycemia. Among those, two-thirds were on insulin (46/69, 66.7%).

### Main analysis

**Fig. 2:**
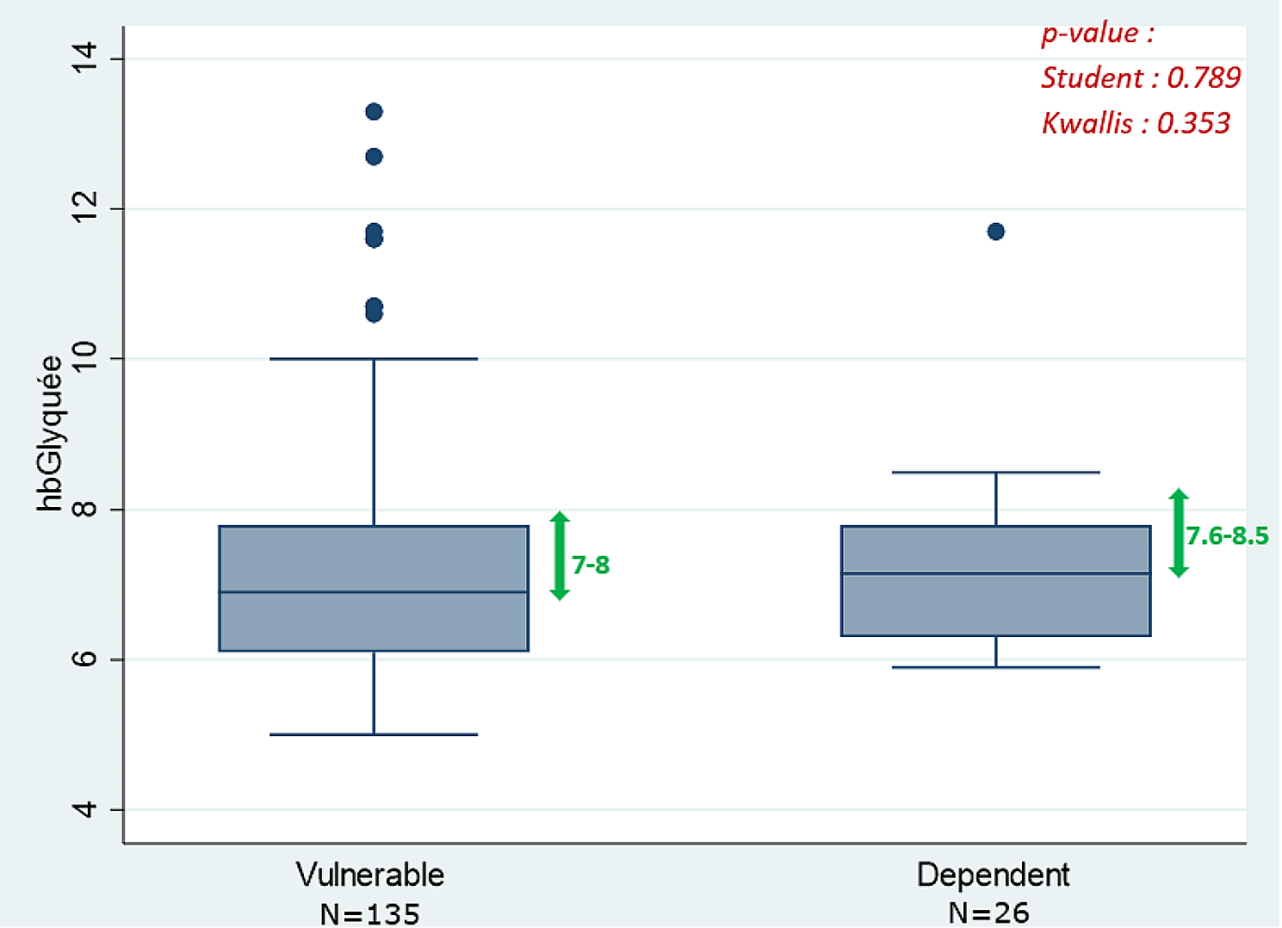
Boxplot of the glycated hemoglobin according to geriatric health profile (vulnerable and dependent). Greens arrows provide the range of HbA1c values defined as target for each group of patients

Overall, 29.8% of the patients had a HbA1c within the defined target according to their health profile (vulnerable and dependent), 54.0% were considered as *overtreated*, and 16.2% as *undertreated*(Table 1). Results specific to dependent patients showed that about one third (9/26) were within the defined HbA1c target but more than 60% (16/26) *overtreated* (Fig. 2). A further analysis differentiating the periods from 2013 to 2016 and from 2017 to 2020 showed an upward trend in the proportion of *overtreated* patients (47.2% vs. 62.5%) and a downward trend in the proportions of *undertreated* (18.0% vs. 13.9%) and *adequately* treated (34.8% vs. 23.6%) patients, respectively.

According to the bivariable analysis, receiving insulin or a treatment with a high risk of hypoglycemia were associated with a decreased risk to be *overtreated* and an increased risk to be *undertreated*. Patients considered as *undertreated* tended to have moderate to severe leukoencephalopathy, multiples comorbidities and to receive formal in-home care services compared to *overtreated* and to *adequately* treated patients but the results were not statistically significant (Table 1). In contrast, neither age nor the severity of cognitive or functional impairment were associated with a risk of being *under-* or *overtreated*.

In multinomial regression, the type of diabetes treatment was the only factor that remained associated with the risk of *under-* and *overtreatment* after adjusting for potential confounders. Receiving a treatment with a high-risk of hypoglycemia was associated both to a lower risk of being *overtreated* (_adj_RRR = 0.28, CI: [0.13–0.62], *p* = .002) and to an increased risk of being *undertreated* (_adj_RRR = 11.80, CI: [2.41–57.80], *p* = .002). The sensitivity analysis using the “Choosing Wisely” definition of overtreatment showed similar results (see appendix).

### Brain morphometry

Patients were further selected if they had a documented MRI (*N* = 83) within 3 weeks before or 18 months after the consultation (median 47.5 days after consultation). Only 12 patients were considered as *undertreated* and were excluded from this subgroup analysis, leaving a final sample of *N* = 71.

This analysis compared the subsets of patients considered as *overtreated* (*N* = 46) to those (*N* = 25) considered as *adequately* treated (HbA1c value “in the target” according to health risk profile).

**Fig. 3:**
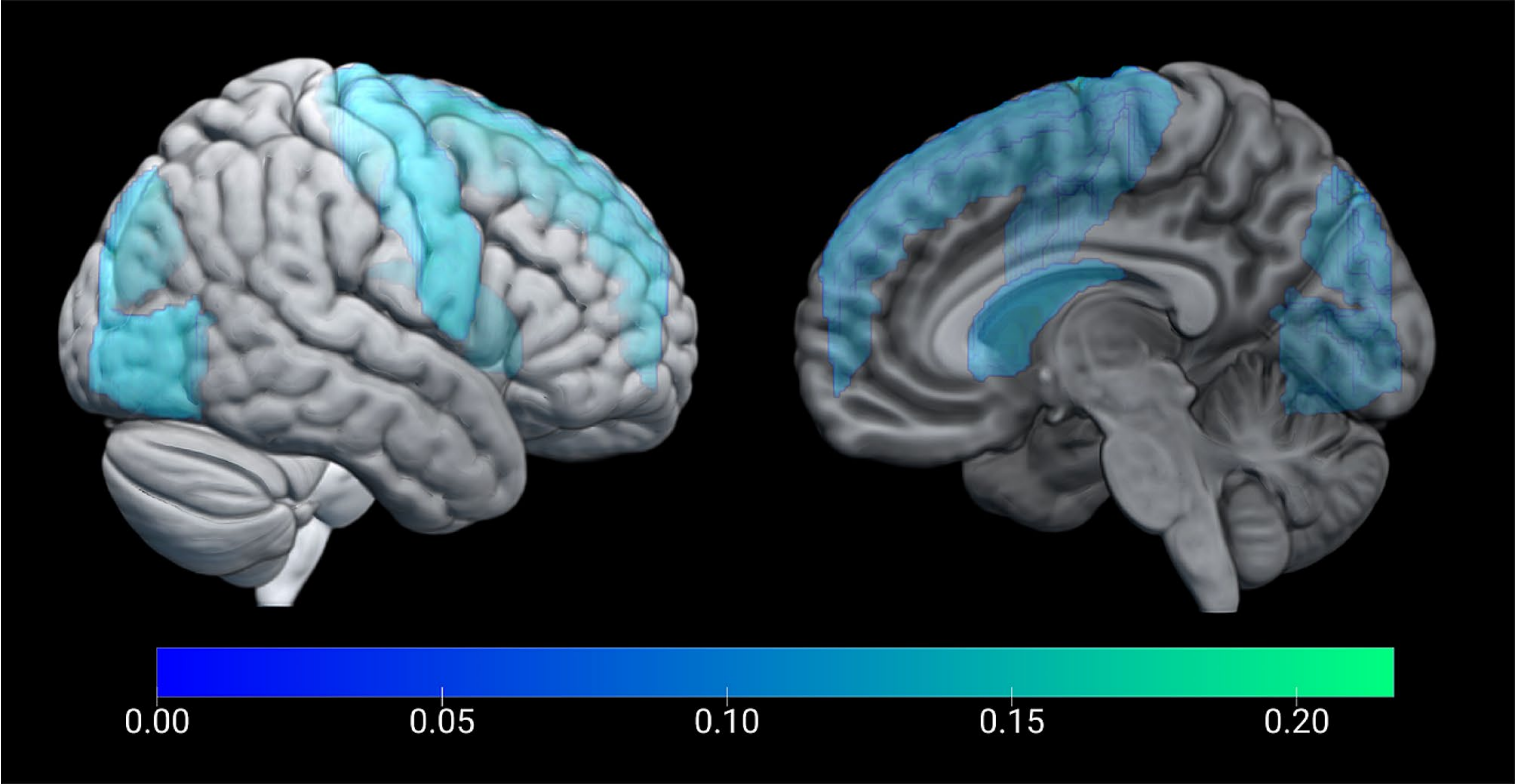
Overlay of the statistical results on a standard brain – outer and inner hemispheric surface. Regions-of-interest weighted by their negative β-coefficients are presented in shades of blue (*p* < .05)

Adjusting for age, sex, treatments associated with a high risk of hypoglycemia, and hypertension, our analysis revealed associations between overtreatment and reduced grey matter volume in multiple regions, as detailed in Fig. 3. Specifically, we observed significant reductions in:

• The caudate (adjusted β coefficient: -0.217; 95% CI: [-0.416, -0.018]; *p* = .033),
• Precentral gyri (adjusted β coefficient: -0.277; 95% CI: [-0.482, -0.073]; *p* = .009),
• Superior frontal gyri (adjusted β coefficient: -0.244; 95% CI: [-0.458, -0.030]; *p* = .026),
• Calcarine cortex (adjusted β coefficient: -0.193; 95% CI: [-0.386, -0.001]; *p* = .049),
• Superior occipital gyri (adjusted β coefficient: -0.291; 95% CI: [-0.521, -0.061]; *p* = .014),
- Inferior occipital gyri (adjusted β coefficient: -0.236; 95% CI: [-0.456, -0.015], *p* = .036).

The supplementary analysis that used the « Choosing Wisely » campaign criteria for diabetes overtreatment in older persons provided similar results in three of the six brain regions identified in the main analysis (inferior and superior occipital gyri, precentral gyri), whereas volume reductions for the caudate, the superior frontal gyri, and the calcarine cortex were still present but did not achieve statistical significance (see appendix). In contrast, several additional regions with significant volume reductions in overtreated participants were identified (cuneus, middle occipital gyri, opercular part of the inferior frontal gyrus).

## Discussion

One of the principal findings of our research is that over half of the cognitively impaired patients in our study were subject to overtreatment for diabetes. This underscores the challenges in applying current treatment guidelines to older, vulnerable diabetic populations, especially if they are not on a treatment with a high-risk of hypoglycemia. These results observed in this specific population of older adults consulting an academic memory clinic extend finding from studies in different population such as community-dwelling or hospitalized older persons, and residents in long-term care facilities [21–23]. Another significant contribution of the present study is to highlight the concerning rising trend in the proportion of patients experiencing overtreatment. This observation aligns with recent findings from a multicentric study across Germany, Austria, Switzerland, and Luxembourg, suggesting a broader pattern of concern [36], even though some other studies conducted in Japan [24] and in Poland [37] did not report a similar trend.

Contrary to our initial hypothesis, the health profile, and the degree of cognitive or functional impairment were not predictive of over- or undertreatment. But our results highlight that patients receiving a treatment with a high-risk of hypoglycemia were more likely to be undertreated, suggesting that this specific risk was better taken into consideration than the risk associated with the intensity of treatment, as reflected by HbA1c. This finding may reflect local practice [38] but may also result from a particular caution in recent years concerning such medications. This was highlighted by the *2019 Endocrine Society’ Guidelines* that added to the health profile a distinction between patients on treatment with a high-risk of hypoglycemia or not [20], an addition that strengthens and further clarifies the definition of diabetes’ overtreatment. In the present study, we used the *ADA*’s *2020* definition of diabetes overtreatment that was a standard at the time of the study. Although this definition permits to better individualizing HbA1c targets, a better definition needs to be developed for older adults that further take into account the type of treatment and its adverse effects, some geriatric syndromes (polymedication, undernutrition, frailty) and the patient’s wishes [39]. This appears especially warranted in the context of the growing enthusiasm for SGLT-2 inhibitors and GLP-1 analogues, but for which studies in frail geriatric patients are lacking [40].

To our knowledge, the present study is the first to use voxel-based morphometry to evaluate the association of type 2 diabetes overtreatment in a geriatric population with neurocognitive disorder. It provides unique information on potential grey matter volume loss in several brain regions in this group of patients. Type 2 diabetes can induce a grey matter loss of volume in defined brain regions, particularly in the frontal and temporal lobes [41, 42], and in the subcortical structures like hippocampus, thalamus, caudate, putamen, and nucleus accumbens [43]. Intensive treatment with a low HbA1c target seems even to provide a slight protection against grey matter volume loss in some of these areas in younger diabetic people [41]. However, the regions affected on neuroimaging in overtreated older patients with diabetes in the present study differ from regions affected by type 2 diabetes mellitus *per se.* In particular, the limbic and sensorimotor systems as well as the posterior area appear at particularly increased risk, in contrast to the hippocampus. Interestingly, several previous studies showed that the posterior area and, to a lesser extent, the caudate, are the most frequently regions affected in case of neonatal hypoglycemia [44–46]. As *Lee et al.* [25], we also observed a significant loss of grey matter volume in the frontal lobe and similarly we found no difference in hippocampal volume. Likely, this lack of association resulted from the sample population of the present study that was composed solely of patients with neurocognitive disorder. The hypothesis is that the impact of the dementia itself on the hippocampus precluded to show an additional volume loss from overtreatment-induced hypoglycemia events. A different vulnerability of the brain to hypoglycemia in younger versus older patients could be hypothesized. Most of these latter patients already present microangiopathic damages [5] and may have a greater sensitivity to overtreatment-induced hypoglycemia events [47].

The study is subject to several limitations that merit consideration. Firstly, the absence of cumulative data on glycemic control poses a challenge to fully understanding the impact of HbA1c levels on the outcomes observed. HbA1c can be affected by the turnover of red blood cells, and should therefore be used with caution in older patients because of their greater number of comorbidities [48]. The future, with continuous glucose monitoring, will probably allow to obtain more reliable data [49]. Second, the small number of undertreated patients did not allow to examine the specific association between elevated HbA1c and brain gray matter volume and could only show a non-significative trend in the bivariable analysis for the association of undertreatment and the number of comorbidities or presence of homecare. Third, due to the absence of available HBA1c in the defined time frame, a large proportion of women could not be included. This could have influenced our results because there are sex differences in prevalence of type 2 diabetes et dementia, with a larger proportion of men suffering from type 2 diabetes. This could lead to earlier onset of dementia in men as shown in a recent study [50], maybe because of more premature microvascular disease [51]. Fourth, the MRI subgroup represented less than half of the cohort recruited. Thus, a selection bias cannot be completely ruled out, even if the MRI analysis has been adjusted for age, gender, treatments associated with a high risk of hypoglycemia, and hypertension. Although the two groups were broadly similar, patients without MRI performed within the study timeframe showed more cerebrovascular involvement and more comorbidity (see appendix). Fifth, the study was performed in a single-center and generalization to other health care environment should be cautious, but prevalence of type 2 diabetes in Switzerland is comparable to the neighboring countries [38] and dementia probably too [52]. Finally, the relatively long inclusion period (2013 to 2020) implies several changes in guidelines about the management of older patients with diabetes. Caution is therefore necessary when interpretating certain aspects, noteworthy overtreatment.

However, this study has several strengths such as the clinically well-defined cohort (older patients with type 2 diabetes and a neurocognitive disorder followed in an academic memory center), thorough investigation of the patients ‘record by the single dedicated investigator, as well as the classification of the patients according to their *ADA’s* health profile, which permit to distinguish vulnerable from dependent patients and therefore provide more detailed and specific information for each in this heterogeneous population and who do not present the same care challenges. The sensitivity analysis based on “the Choosing Wisely” definition of overtreatment further underlines the robustness of our results in showing similar results in terms of risk factors for overtreatment and brain regions affected (either identical or close to each other). Indeed, regions that were identified only in the “Choosing Wisely” analysis were all closely situated to regions with significant volume reductions in the initial analysis, the posterior zone seeming particularly affected.

The present study also highlights several areas to improving future studies, such as reaching consensus on a more integrative definition of overtreatment in older adults that takes into account the type of treatment and its adverse effects, in addition to these patient’s health status, functional profile, and wishes. Similarly, the use of more valid data about hypoglycemia is desirable, for example using continuous glucose monitoring. This step will be critical to better understand the vulnerability of specific cerebral regions to repeated hypoglycemia or hyperglycemia. Finally, the investigation of the potential protective effect on cognition of new molecules such as SGLT-2 inhibitors and GLP-1 receptor agonists is also warranted.

## Conclusion

This study suggests that a large proportion of older patients with type 2 diabetes remain subjected to excessively intensive treatment. Moreover, the results highlight a significative association with brain volume loss in several brain regions. Overall, these findings strongly suggest that major efforts are still needed to better inform older patients with diabetes, proxies, and their healthcare providers about the risk associated with inadequate treatment. Future studies will be able to complete these results by studying the potential protective effect on cognition of the new molecules, including SGLT-2 inhibitors and GLP-1 receptor agonists. Furthermore, a better understanding of the specific cerebral regions impacted by repeated hypoglycemia will help in the de-prescription process in this vulnerable population.

### Electronic supplementary material

Below is the link to the electronic supplementary material.

Supplementary Material 1

Supplementary Material 2

## Data Availability

The datasets used and analysed during the current study are available from the corresponding author on reasonable request.
