# Supplementary material for: Diabetes mellitus in older persons with neurocognitive disorder: overtreatment prevalence and associated structural brain MRI findings
Source: BMC Geriatr. 2024 May 14;24:427. doi: 10.1186/s12877-024-05025-x (PMC11095019; doi:10.1186/s12877-024-05025-x)
Supplement: Supplementary file 1 — Supplementary Material 1 [file 12877_2024_5025_MOESM1_ESM.docx]

***Appendix***

1. **Sensitivity Analysis with the “Choosing Wisely” definition of diabetes overtreatment**

The “Choosing Wisely” definition is the following: “Reasonable glycemic targets would be 7.0 – 7.5% in healthy older adults with long life expectancy, 7.5 – 8.0% in those with moderate comorbidity and a life expectancy < 10 years, and 8.0 – 9.0% in those with multiple morbidities”

- 1. **Bivariable analysis comparing the characteristics of under-, adequately and over-treated patients**

|  | | Treatment adequation | | |  |
| --- | --- | --- | --- | --- | --- |
|  | **All**  N=161  (100.0%) | **Undertreated**  N=26  (16.2%) | **Adequate**  N=25 (15.5%) | **Overtreated**  N=110 (68.3%) | **p-value*** |
| Female Sex | 53 (32.9) | 7 (26.9) | 12 (48.0) | 34 (30.9) | .202 |
| Age  Median  [range] | 76.8  [60.8-93.3] | 75.6  [62.7-86.2] | 77.3  [61.5-93.3] | 76.7  [60.8-91.6] | .635 |
| Health status  Vulnerable  Dependent | 135 (83.9)  26 (16.2) | 25 (96.2)  1 (3.9) | 20 (80.0)  5 (20.0) | 90 (81.8)  20 (18.2) | .152 |
| Living alone | 54 (33.8) | 12 (46.2) | 9 (36.0) | 33 (30.3) | .296 |
| Homecare present | 61 (38.4) | 14 (56.0) | 8 (32.0) | 39 (35.8) | .134 |
| ≥ 3 comorbidities | 90 (55.9) | 20 (76.9) | 13 (52.0) | 57 (51.8) | .062 |
| Katz Basic ADL^†^ <4 | 11 (6.8) | 1 (3.9) | 2 (8.0) | 8 (7.3) | .900 |
| Lawton Instrumental ADL ^‡^ <6 | 70 (43.5) | 12 (46.2) | 10 (40.0) | 48 (43.6) | .905 |
| Etiology of the cognitive disorder  Alzheimer’s disease  Vascular dementia  Other  Missing | 52 (32.5)  65 (40.6)  43 (26.9)  1 | 7 (28.0)  11 (44.0)  7 (28.0)  1 | 10 (40.0)  9 (36.0)  6 (24.0)  0 | 35 (31.8)  45 (40.9)  30 (27.3)  0 | .924 |
| CDR ^§^ >0.5 | 66 (41.0) | 10 (38.5) | 9 (36.0) | 47 (42.7) | .793 |
| Leukoencephalopathy  Moderate/severe  Light  Missing | 67 (44.1)  85 (55.9)  9 | 17 (68.0)  8 (32.0)  1 | 10 (43.5)  13 (56.5)  2 | 40 (38.5)  64 (61.5)  6 | .028 |
| Insulin in the treatment | 46 (28.6) | 21 (80.8) | 8 (32.0) | 17 (15.5) | <.001 |
| Treatment with a high risk of hypoglycemia | 69 (42.9) | 24 (92.3) | 14 (56.0) | 31 (28.2) | <.001 |

* p-value from Pearson chi-squared test or Fisher exact test (categorical variables), and Student’s t-test or Wilcoxon’s test (continuous variables)

† Katz Basic Activities of Daily Living (BADL): includes bathing, dressing, going to the WC, transferring, maintaining continence, eating, score ranges from 0 to 6 with higher scores indicating better function (29)

‡ Lawton Instrumental Activities of Daily Living (IADL): includes using the phone, managing the finances, managing the medication, preparing meals, doing the laundry, cleaning, shopping, and using the transportation; Score range from 0 to 8 with higher scores indicating better function (30)

§ CDR: calculated on the basis of six different cognitive and behavioral domains such as memory, orientation, judgment and problem solving, community affairs, home and hobbies performance, and personal care. Scale of 0–3: no dementia (CDR = 0), very mild cognitive impairment (CDR = 0.5), mild dementia (CDR = 1), moderate (CDR = 2), and severe dementia (CDR = 3) (53)

- 1. **Multinomial regression**

The type of diabetes treatment is the only factor that remains associated with the risk of *under-* and *overtreatment* after adjusting for potential confounders.

Receiving a treatment with a high-risk of hypoglycemia was associated both to a lower risk of being *overtreated* (_adj_RRR = 0.29, CI: [0.11-0.75], p=.011) and to an increased risk of being *undertreated* (_adj_RRR = 8.84, CI: [1.61-48.46], p=.012).

- 1. **MRI Analysis**

The MRI-analysis compared the subsets of patients considered as *overtreated* (N=46) to those (N=25) considered as *adequately* treated. Adjusting for age, sex, treatments associated with a high risk of hypoglycemia, and hypertension, our analysis revealed associations between overtreatment and reduced grey matter volume in:

- Cuneus (adjusted β coefficient: -0.217; 95% CI: [-0.433, -0.002]; p =.048),
- Superior occipital gyri (adjusted β coefficient: -0.331; 95% CI: [-0.566, -0.096]; p =.006),
- Inferior occipital gyri (adjusted β coefficient: -0.225; 95% CI: [-0.451, -0.000], p=.050),
- Middle occipital gyri (adjusted β coefficient: -0.246; 95% CI: [-0.456, -0.035], p=.023),
- Precentral gyri (adjusted β coefficient: -0.209; 95% CI: [-0.406, -0.011]; p = .039),
- Opercular part of the inferior frontal gyrus (adjusted β coefficient: -0.209; 95% CI: [-0.383, -0.036]; p = .019),

Each reported value reflects the adjustment for predefined covariates.

1. **Analysis comparing the characteristics of included patients to those of patients excluded because of lacking HbA1C value within the defined timeframe**
   1. **Bivariable analysis comparing the characteristics of the included patients to those of patients excluded because of lacking HbA1C value within the defined timeframe**

|  | All patients screened  N=472 (100.0%) | HbA1c available within the timeframe | |  |
| --- | --- | --- | --- | --- |
|  |  | **No**  N=311  (65.9%) | **Yes**  N=161  (34.1%) | **P-value*** |
| Female Sex | 198 (42.0) | 145 (46.6) | 53 (32.9) | 0.004 |
| Age  Median  [range] | 76.0  60.0//93.3 | 76.0  60.0//93.0 | 76.8  60.8//93.3 | 0.513 |
| Katz ADL ^†^ <4  Missing | 15 (5.4)  193 | 4 (3.4)  193 | 11 (6.8)  0 | 0.285 |
| Lawton’s IADL ^‡^ <6  Missing | 118 (42.1)  192 | 48 (40.3)  192 | 70 (43.5)  0 | 0.599 |
| Etiology of the cognitive disorder  Alzheimer’s disease  Vascular dementia  Other | 186 (39.5)  164 (34.8)  121 (25.7) | 134 (43.1)  99 (31.8)  78 (25.1) | 52 (32.5)  65 (40.6)  43 (26.9) | 0.065 |
| CDR ^§^ >0.5  Missing | 98 (43.8)  248 | 32 (50.8)  248 | 66 (41.0)  0 | 0.184 |

* p-value from Pearson chi-squared test or Fisher exact test (categorical variables), and Student’s t-test or Wilcoxon’s test (continuous variables)

† Katz Basic Activities of Daily Living (BADL): includes bathing, dressing, going to the WC, transferring, maintaining continence, eating, score ranges from 0 to 6 with higher scores indicating better function^26^

‡ Lawton Instrumental Activities of Daily Living (IADL): includes using the phone, managing the finances, managing the medication, preparing meals, doing the laundry, cleaning, shopping, and using the transportation; Score range from 0 to 8 with higher scores indicating better function^27^

§ CDR: calculated on the basis of six different cognitive and behavioral domains such as memory, orientation, judgment and problem solving, community affairs, home and hobbies performance, and personal care. Scale of 0–3: no dementia (CDR = 0), very mild cognitive impairment (CDR = 0.5), mild dementia (CDR = 1), moderate (CDR = 2), and severe dementia (CDR = 3)^43^

1. **Analysis comparing the characteristics of patients included** **with and without MRI subgroup to recruited cohort**
   1. **Bivariable analysis comparing the characteristics of included patients with and without MRI data within the defined timeframe**

|  | All patients included  N=161  (100.0%) | MRI available within the timeframe | |  |
| --- | --- | --- | --- | --- |
|  |  | **Yes**  N=83  (51.6%) | **No**  N=78  (48.5%) | **p-value*** |
| Female Sex | 53 (32.9) | 25 (30.1) | 28 (35.9) | .436 |
| Age  Median  [range] | 76.8  [60.8-93.3] | 76.3  [60.8-90.9] | 77.2  [61.3-93.3] | .655 |
| Health status  Vulnerable  Dependent | 135 (83.9)  26 (16.2) | 73 (88.0)  10 (12.1) | 62 (79.5)  16 (20.5) | .145 |
| Living alone | 54 (33.8) | 24 (29.3) | 30 (38.5) | .219 |
| Homecare present | 61 (38.4) | 26 (32.1) | 35 (44.9) | .098 |
| ≥ 3 comorbidities | 90 (55.9) | 40 (48.2) | 50 (64.1) | .042 |
| Katz Basic ADL^†^ <4 | 11 (6.8) | 5 (6.0) | 6 (7.7) | .761 |
| Lawton Instrumental ADL ^‡^ <6 | 70 (43.5) | 33 (39.8) | 37 (47.4) | .326 |
| Etiology of the cognitive disorder  Alzheimer’s disease  Vascular dementia  Other  Missing | 52 (32.5)  65 (40.6)  43 (26.9)  1 | 35 (42.7)  28 (34.2)  19 (23.2)  1 | 17 (21.8)  37 (47.4)  24 (30.8)  0 | .019 |
| CDR ^§^ >0.5 | 66 (41.0) | 33 (39.8) | 33 (42.3) | .742 |
| Leukoencephalopathy  Moderate/severe  Light  Missing | 67 (44.1)  85 (55.9)  9 | 28 (34.2)  54 (65.9)  1 | 39 (55.7)  31 (44.3)  8 | .008 |
| Insulin in the treatment | 46 (28.6) | 21 (25.3) | 25 (32.1) | .343 |
| Treatment with a high risk of hypoglycemia | 69 (42.9) | 34 (41.0) | 35 (44.9) | .617 |

* p-value from Pearson chi-squared test or Fisher exact test (categorical variables), and Student’s t-test or Wilcoxon’s test (continuous variables)

† Katz Basic Activities of Daily Living (BADL): includes bathing, dressing, going to the WC, transferring, maintaining continence, eating, score ranges from 0 to 6 with higher scores indicating better function^26^

‡ Lawton Instrumental Activities of Daily Living (IADL): includes using the phone, managing the finances, managing the medication, preparing meals, doing the laundry, cleaning, shopping, and using the transportation; Score range from 0 to 8 with higher scores indicating better function^27^

§ CDR: calculated on the basis of six different cognitive and behavioral domains such as memory, orientation, judgment and problem solving, community affairs, home and hobbies performance, and personal care. Scale of 0–3: no dementia (CDR = 0), very mild cognitive impairment (CDR = 0.5), mild dementia (CDR = 1), moderate (CDR = 2), and severe dementia (CDR = 3)^43^

1. **Brain subregions used for the MRI analysis are the following:**

Accumbens, caudate, pallidum, putamen, thalamus proper, amygdala, hippocampus, entorhinal area, parahippocampal gyrus, calcarine cortex, lingual gyrus, fusiform gyrus, cuneus, inferior occipital gyrus, occipital pole, occipital fusiform gyrus, middle occipital gyrus, superior occipital gyrus, angular gyrus, superior parietal lobule, anterior cingulate gyrus, posterior cingulate gyrus, middle cingulate gyrus, precuneus, central operculum, frontal operculum, postcentral gyrus medial segment, postcentral gyrus, supramarginal gyrus, parietal operculum, planum temporale, anterior insula, posterior insula, planum polare, precentral gyrus medial segment, precentral gyrus, supplementary motor cortex, frontal pole, medial orbital gyrus, medial frontal cortex, subcallosal area, gyrus rectus, lateral orbital gyrus, anterior orbital gyrus, posterior orbital gyrus, basal forebrain, middle frontal gyrus, superior frontal gyrus, triangular part of the inferior frontal gyrus, orbital part of the inferior frontal gyrus, opercular part of the inferior frontal gyrus, superior frontal gyrus medial segment, temporal pole, middle temporal gyrus, transverse temporal gyrus, inferior temporal gyrus, superior temporal gyrus, cerebellum exterior, cerebellar vermal lobules I to V, cerebellar vermal lobules VI-VII, and cerebellar vermal lobules VIII to X.

1. **Type of glucose-lowering medication in the analyzed population**
