# Supplementary material for: Diabetes mellitus in older persons with neurocognitive disorder: overtreatment prevalence and associated structural brain MRI findings
Source: BMC Geriatr. 2024 May 14;24:427. doi: 10.1186/s12877-024-05025-x (PMC11095019; doi:10.1186/s12877-024-05025-x)
Supplement: Supplementary file 2 — Supplementary Material 2 [file 12877_2024_5025_MOESM2_ESM.docx]

**List of abbreviations**

Glycated hemoglobin (HbA1c)

Magnetic resonance imaging (MRI)

Clinical Dementia Rating (CDR)

Electronic health record (EHR)

*The American Diabetes Association’s* (ADA)

Cohort Leenaards Memory and Neurosciences (CLEMENS)

Basic activities of daily living (BADL)

Instrumental activities of daily living (IADL)

Glomerular filtration rate (GFR)

Body mass index (BMI)

Analysis of variance (ANOVA)
